# Hole Transfer in Open Carbynes

**DOI:** 10.3390/ma13183979

**Published:** 2020-09-08

**Authors:** Constantinos Simserides, Andreas Morphis, Konstantinos Lambropoulos

**Affiliations:** Department of Physics, National and Kapodistrian University of Athens, Panepistimiopolis, Zografos, GR-15784 Athens, Greece; amorphis@phys.uoa.gr (A.M.); klambro@phys.uoa.gr (K.L.)

**Keywords:** charge (hole) transfer, Real-Time Time-Dependent Density Functional Theory (RT-TDDFT), Tight-Binding (TB), carbynes, cumulenes, polyynes

## Abstract

We investigate hole transfer in open carbynes, i.e., carbon atomic nanowires, using Real-Time Time-Dependent Density Functional Theory (RT-TDDFT). The nanowire is made of *N* carbon atoms. We use the functional B3LYP and the basis sets 3-21G, 6-31G*, cc-pVDZ, cc-pVTZ, cc-pVQZ. We also utilize a few Tight-Binding (TB) wire models, a very simple model with all sites equivalent and transfer integrals given by the Harrison ppπ expression (TBI) as well as a model with modified initial and final sites (TBImod) to take into account the presence of one or two or three hydrogen atoms at the edge sites. To achieve similar site occupations in cumulenes with those obtained by converged RT-TDDFT, TBImod is sufficient. However, to achieve similar frequency content of charge and dipole moment oscillations and similar coherent transfer rates, the TBImod transfer integrals have to be multiplied by a factor of four (TBImodt4times). An explanation for this is given. Full geometry optimization at the B3LYP/6-31G* level of theory shows that in cumulenes bond length alternation (BLA) is not strictly zero and is not constant, although it is symmetrical relative to the molecule center. BLA in cumulenic cases is much smaller than in polyynic cases, so, although not strictly, the separation to cumulenes and polyynes, approximately, holds. Vibrational analysis confirms that for *N* even all cumulenes with coplanar methylene end groups are stable, for *N* odd all cumulenes with perpendicular methylene end groups are stable, and the number of hydrogen atoms at the end groups is clearly seen in all cumulenic and polyynic cases. We calculate and discuss the Density Functional Theory (DFT) ground state energy of neutral molecules, the CDFT (Constrained DFT) “ground state energy” of molecules with a hole at one end group, energy spectra, density of states, energy gap, charge and dipole moment oscillations, mean over time probabilities to find the hole at each site, coherent transfer rates, and frequency content, in general. We also compare RT-TDDFT with TB results.

## 1. Introduction

Carbynes are one-atom-thick, carbon-based, ideal nanowires. Simplistically, carbon atoms adopt sp hybridization. Let us assume that the chain is laid along the *z*-axis. Then, each carbon atom is connected with a $sp_{z}sp_{z}\sigma$ bond to its previous and next neighbor and has one $p_{x}$ and one $p_{y}$ electron that form two, energy-degenerate, π-stacks. $p_{i}$, i=x,y,z, means $2p_{i}$ for carbon atoms. Usually, we state that there are two “possible” types of carbynes, cumulenic and polyynic. In cumulenic carbynes, the bond length alternation (BLA), the difference between the distances of consecutive carbon atoms, is zero, while, in polyynic carbynes, BLA is not zero; typically we have an alternation of short (*s*) and long (*l*) bonds; hence we obtain polyynic *sl* (*ls*) molecules having sequences of short-long-... (long-short-...) bonds. This simplistic description neglects the presence of end groups that is, depending on the particular configuration, CH_3_– or CH_2_– or CH–.

We discriminate between the terms transport and transfer: transport implies application of electric voltage between electrodes connected at the ends of the system; transfer implies that an extra electron or hole, created, e.g., by reduction or oxidation at a certain site, moves to more favorable sites, without application of external voltage. Experimental [1,2,3,4] as well as theoretical work [5,6,7,8,9] on charge transport in carbynes has been accomplished. However, it seems that there is no experiment of charge transfer along these molecules, apart from a very recent work [9]. In this work, the authors study terminated carbon chains of two to eight carbon atoms, and report that the increase of the wire length alters its function from an electron donor to an electron acceptor, but no transfer rate was reported. Experience from experiments of charge transfer along DNA shows that possibly a direct approach for this aim could be time-resolved spectroscopy [10,11,12,13,14,15,16].

In Ref. [17] we studied theoretically and computationally hole transfer in cumulenic and polyynic carbynes, and arrived to several conclusions, summarized in this paragraph. By using several basis sets of increasing size, we demonstrated the convergence of our results. In most cases, the smallest basis set of sufficient quality was cc-pVTZ; cc-pVQZ was, of course, better, yet the computational cost was higher [17]. From DFT simulations on neutral molecules we obtained that for the ground-state energy, $E_{\mathrm{GS}}$, it holds that

$$\begin{array}{cc} E_{\mathrm{GS}}^{\mathrm{polyynic}\mathrm{ls}}=E_{\mathrm{GS}}^{\mathrm{polyynic}\mathrm{sl}}\overset{\mathrm{slightly}}{>}E_{\mathrm{GS}}^{\mathrm{cumulenic}}, & \mathrm{for}N\mathrm{odd},\;\mathrm{and}\\ E_{\mathrm{GS}}^{\mathrm{polyynic}\;}>E_{\mathrm{GS}}^{\mathrm{cumulenic}}>E_{\mathrm{GS}}^{\mathrm{polyynic}\mathrm{ls}}, & \mathrm{for}N\mathrm{even}. \end{array}$$

The Density Functional Theory (DFT) simulations showed that cumulenic molecules with odd *N* in which the methylene groups are perpendicular (*pe*) have lower $E_{\mathrm{GS}}$ compared the respective ones in which the methylene groups are coplanar (*co*) [17]. As expected, all molecules in which a hole was inserted gave systematically larger energies than the respective neutral ones, otherwise, neutral molecules would not be stable. A simple Tight-Binding (TB) wire model with equal on-site energies (TBI) as well as another one with modified on-site energies and hopping integrals to account for the CH_n_– end groups (TBImod) reproduced approximately the magnitude of the energy gap in the polyynic case. The DFT calculations showed that, due to the presence of end groups, there exists a cumulenic energy gap, too, smaller than the polyynic one [17]. The Real-Time Time-Dependent Density Functional Theory (RT-TDDFT) calculations showed that the mean over time probabilities to find the hole at various sites (site occupations) converged with increasing the size of the basis set. TBImod agreed with the mean over time probabilities (site occupations) RT-TDDFT predicted, for cumulenic molecules. The site occupations of polyynic *sl* (i.e., starting with shorter bond length) and of polyynic *ls* (i.e., starting with longer bond length) molecules were different than the cumulenic ones, and the simplistic TBImod model could qualitatively explain the RT-TDDFT trends [17]. However, TBImod (and TBI) predicted charge oscillations that were approximately four times slower than the RT-TDDFT ones. A simple Fast Fourier Transform (FFT) analysis of dipole moment oscillations, which are independent of the population analysis used, confirmed that fact. Similarly faster were found the coherent transfer rates *k* predicted by RT-TDDFT compared to those predicted by TBImod (and TBI) [17]. k(N) or lnk(lnN) converged increasing the basis set. TBImod was, as explained, slower but followed the trend. The trends in the behaviour of k(N) or lnk(lnN) as predicted by RT-TDDFT could be qualitatively explained by TBImod, although oscillations in RT-TDDFT were always faster [17]. We are expecting experiments to obtain coherent transfer rates in carbynes, probably using time-resolved spectroscopy.

In the present article we elaborate our calculations on hole transfer in open carbynes. In Section 2 we discuss bond lengths, structures and vibrational analysis, in Section 3 we present the simple TB models that we use, in Section 4 we delineate RT-TDDFT, in Section 5 we present and discuss our results and in Section 6 we state our conclusions.

## 2. Bond Lengths-Structures-Vibrational Analysis

Table 1 shows the C–C bond lengths in various carbon-based molecules. Bonds in carbynes are among the strongest between carbon atoms. Furthermore, at room temperature, the persistence length of carbynes is about 14 nm [18], that is, approximately 100 carbon atoms (since the bond length is about 0.13 nm). These observations make carbynes promising for applications. Several other interesting properties, such as their tunable band gap, their extreme stiffness and elastic modulus, as well as their high flexibility, justify the broad scientific attention they have attracted.

**Figure 1:**
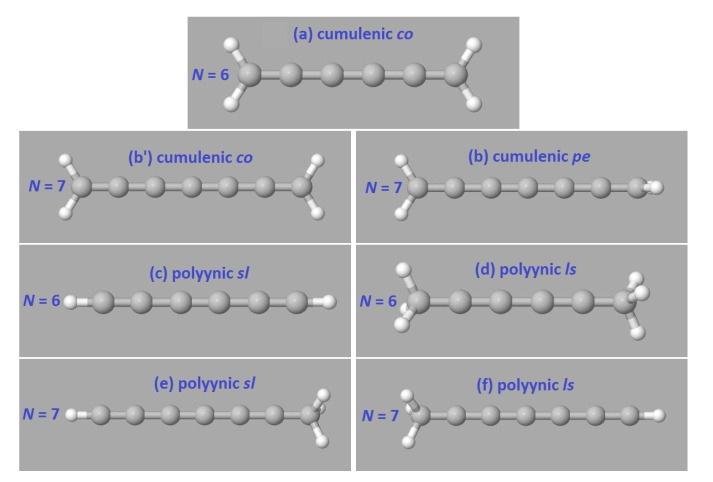
Illustration of cumulenic and polyynic carbynes. By *co* (*pe*) we denote a molecule with coplanar (perpendicular) methylene groups and by *sl* (*ls*) we denote a molecule with short-long-... (long-short-...) sequence of bonds. (**a**) N=6 cumulenic *co*, (**b**’) N=7 cumulenic *co*, (**b**) N=7 cumulenic *pe*, (**c**) N=6 polyynic *sl*, (**d**) N=6 polyynic *ls*, (**e**) N=7 polyynic *sl*, and (**f**) N=7 polyynic *ls*. To study charge transfer, we place a hole initially at the first site, which is made of the first carbon and one or two or three hydrogens. Then, we follow its temporal and spatial evolution. In a simple picture, the first and the last carbons have in (**a**), (**b**’), (**b**) $sp^{2}$, in (**c**) sp, in (**d**) $sp^{3}$, in (**e**) sp and $sp^{3}$, and in (**f**) $sp^{3}$ and sp hybridizations.

In Figure 1, we illustrate cumulenic and polyynic carbynes made of N= 6 and 7 carbon atoms. For *N* even, the cumulene with lower ground state energy is that with coplanar methylene groups, cumulenic *co*, shown in (a). For *N* odd, the cumulene with lower ground state energy is that with perpendicular methylene groups, cumulenic *pe*, shown in (b). For *N* even, placing initially the CH_2_– groups in perpendicular configuration and then optimizing hydrogen atoms, results in CH_2_– groups in coplanar configuration. Therefore, for *N* even, we only include cumulenic *co* molecules in our RT-TDDFT simulations. For *N* odd (even), the ground-state molecule is that with perpendicular (coplanar) end groups [23]. Polyynic *ls* molecules exist with eclipsed and staggered methyl groups for *N* even, with negligibly different ground state energy; in panel (d) we show the staggered configuration.

We have performed vibrational analysis, at the B3LYP/6-31G* level of theory, in NWChem [24] and Gaussian [25] to ensure that the results are correct. Generally, vibrational analysis gives the eigen-frequencies of a molecule’s normal modes. If there is no imaginary eigen-frequency, the geometry corresponds to a minimum of ground-state energy; otherwise, it is either a transition state (one imaginary eigen-frequency) or does not have any particular meaning (more than one imaginary eigen-frequencies). Vibrational analysis has a meaning only in case of full geometry optimization, where the first derivatives of energy with respect to spatial coordinates are zero. Geometry optimization and vibrational analysis must be performed with exactly the same functional and basis set. We have performed vibrational analysis for our cumulenic carbynes of Ref. [17]. With the exception of N= 2 *co*, 3 *pe* and 4 *co*, that had no imaginary eigen-frequencies, the rest had at least one (usually 3–4) imaginary eigen-frequencies. This is because in Ref. [17] we assumed for simplicity that the carbon atoms were held at positions separated by d= 128.2 pm for the cumulenic molecules and $d_{\ell }=$ 130.1 pm, $d_{s}=$ 126.5 pm for the polyynic molecules, following Ref. [22], where the C–C bond lengths of atomic carbon wires had been calculated by DFT. In other words, in Ref. [17] we only optimized the hydrogen positions whereas carbon atoms were kept at fixed positions.

**Figure 2:**
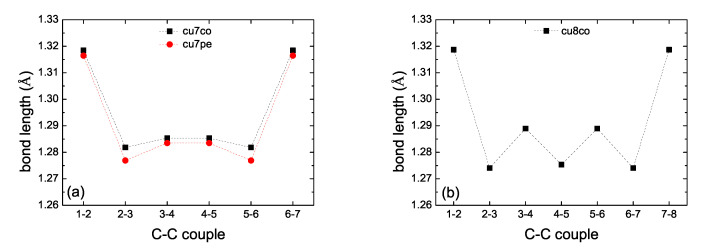

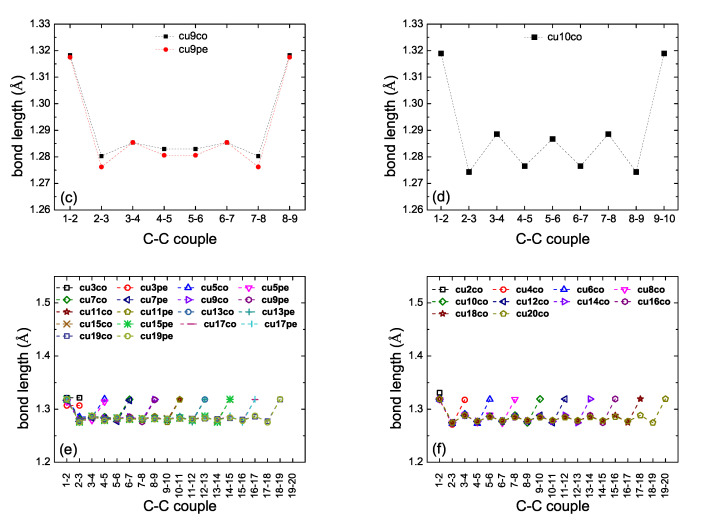
Bond lengths of cumulenic molecules. B3LYP/6-31G* level of theory, without any constraint on the position of atoms. (**a**) N=7, (**b**) N=8, (**c**) N=9, (**d**) N=10, (**e**) *N* odd, (**f**) *N* even.

**Figure 3:**
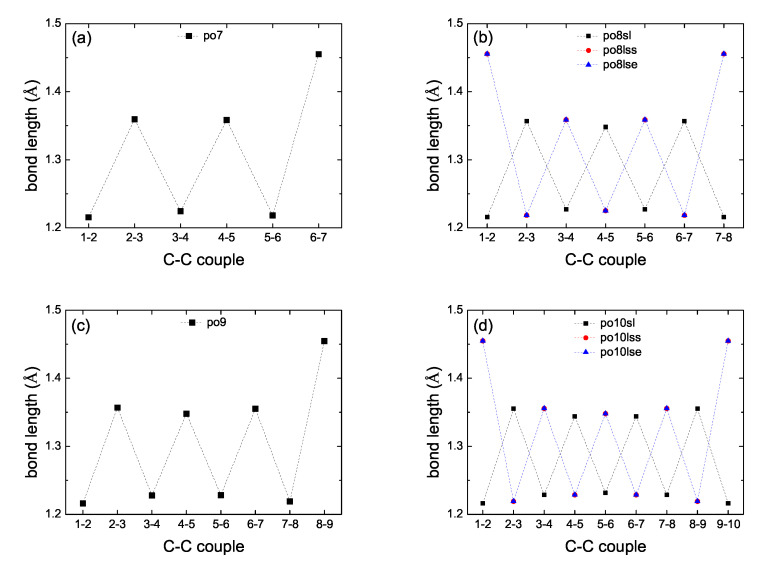

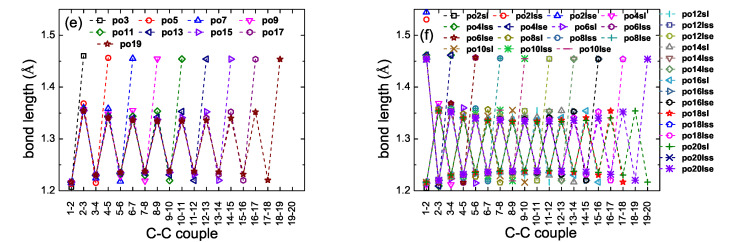
Bond lengths of polyynic molecules. B3LYP/6-31G* level of theory, without any constraint on the position of atoms. (**a**) N=7, (**b**) N=8, (**c**) N=9, (**d**) N=10, (**e**) *N* odd, (**f**) *N* even.

In the present work we performed full geometry optimization of all carbynes, i.e., without keeping fixed the C atoms. For cumulenic carbynes, again, this resulted in two geometries (*co* and *pe*) for *N* odd, but only in one geometry (*co*) for *N* even. Figure 2 shows bond lengths of various cumulenic molecules. The resulting geometries in all cases have longer C–C bonds at the ends (≈132 pm) and C-C bond lengths ≈127 to 129 pm at the middle with alternating longer and shorter bonds. Therefore, the resulting geometry is not strictly cumulenic in the sense that BLA ≠0 and is not constant. However, BLA is symmetrical relative to the molecule center. In Figure 2 panels (a) and (c) we observe that for *N* odd, *pe* molecules have shorter bond lengths, another manifestation that their ground state energy is lower than that of *co* molecules. For polyynic carbynes, geometry optimization again resulted in one geometry, *sl* ≡ *ls*, for *N* odd, but to three geometries for *N* even: *sl*, *lss* and *lse*, the last two denote staggered and eclipsed methyl end groups (the difference between the ground state energy of these last two geometries is almost negligible). Figure 3 shows bond lengths of various polyynic molecules. We observe that the long bonds at the ends are longer than the long bonds at the middle of the molecules. Although Figure 2 panels (e) and (f) and Figure 3 panels (e) and (f) are dense, they certainly show the size of bond length variations in all cumulenic and polyynic cases. If we compare Figure 2 with Figure 3, we realize that BLA in cumulenic cases is much smaller than in polyynic cases, so, although not strictly, the separation to cumulenes and polyynes approximately holds.

**Figure 4:**
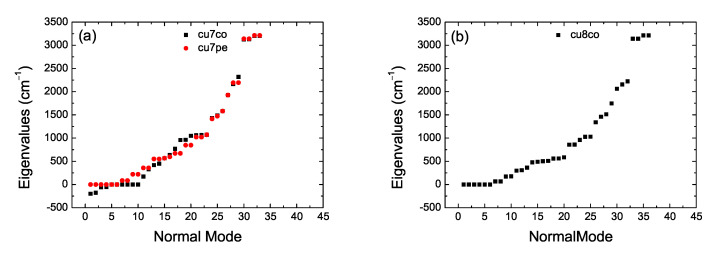

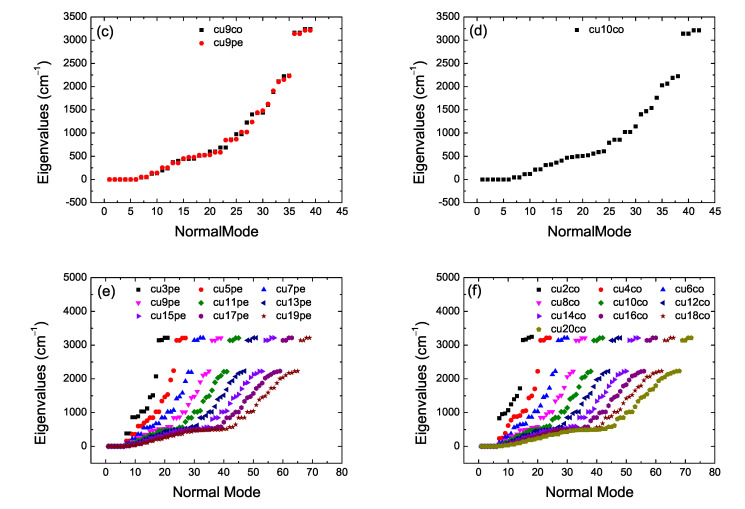
Vibrational analysis of cumulenic molecules. B3LYP/6-31G* level of theory, without any constraint on the position of atoms. (**a**) N=7, (**b**) N=8, (**c**) N=9, (**d**) N=10, (**e**) *N* odd (pe), (**f**) *N* even.

**Figure 5:**
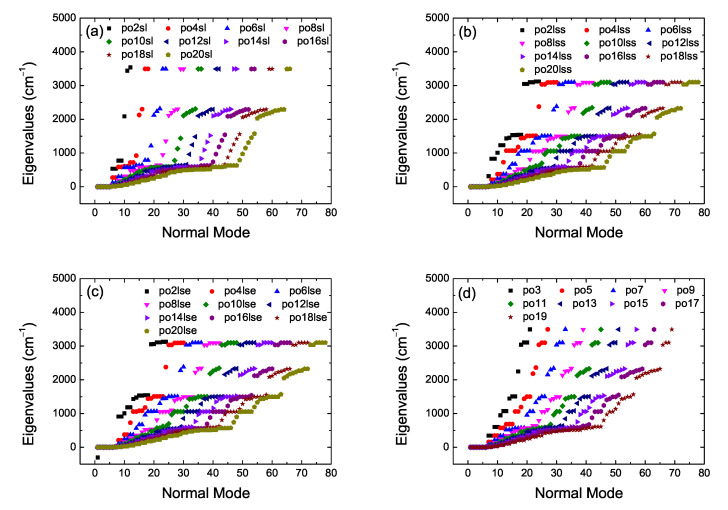
Vibrational analysis of polyynic molecules. B3LYP/6-31G* level of theory, without any constraint on the position of atoms. (**a**) even sl, (**b**) even lss, (**c**) even lse, (**d**) odd.

Let *n* be the number of atoms and *N* the number of carbon atoms (e.g., in cumulenes n=N+4). The number of modes, m=3n, from which 3 are translational modes, and linear (nonlinear) molecules have 2 (3) rotational modes, therefore, the number of vibrational modes is 3n−5 (3n−6). In Table 2 we summarize the situation for our categories. Performing vibrational analysis at the B3LYP/6-31G* level of theory results in all *co* geometries for *N* even being stable and all *pe* geometries for *N* odd being stable, as expected. It seems that also some *co* cumulenes for *N* odd are stable, but, more basis sets might need to be used before coming to a definitive conclusion about this issue. In Figure 4 we present vibrational analysis of cumulenic molecules at the B3LYP/6-31G* level of theory, without any constraint on the position of atoms. We observe the four higher frequencies corresponding to the four hydrogen atoms of cumulenes and that even *N co* and odd *N pe* cumulenic molecules are stable from the point of view of vibrational analysis. In Figure 5 we present vibrational analysis of polyynic molecules at the B3LYP/6-31G* level of theory, without any constraint on the position of atoms. We observe the two (a), six (b), six (c) and 4 (d) higher frequencies corresponding to the number of hydrogen atoms of polyynes for even *N sl*, even *N lss*, even *N lse*, and odd *N* molecules, respectively. Actually, in case (d) we can discern the two different end groups CH– and CH_3_–. The negative values in panel Figure 4a for the N=7 molecule with coplanar methylene groups and in panel Figure 5c for the N=2 *ls* molecule with eclipsed methyl groups are associated with imaginary eigenfrequencies. As explained in Ref. [26]: “If you have optimized to a transition state, or to a higher order saddle point, then there will be some negative frequencies which may be listed before the “zero frequency” modes. (Frequencies which are printed out as negative are really imaginary; the minus sign is simply a flag to indicate that this is an imaginary frequency)”.

Here we would like to mention again [17] that, as has been argued, Peierls distortion [27], due to electron phonon-coupling [28], energetically favors the polyynic configuration [8,18]. Nevertheless, it has been reported, theoretically [29] and experimentally [3], that under no strain, the cumulenic phase is also possible. This has been attributed to the elimination of the Peierls distortion by the zero-point atomic vibrations [29]. Moreover, capping a finite carbyne chain between $sp^{2}$-conjugated end groups with a small number of aromatic units enhances its cumulenic character [7]. As far as we know, DFT does not include an electron phonon-coupling term, hence, although our geometry optimization results and the relevant discussion above are very interesting, we are not certain what the actual situation finally is. Hence, unless otherwise stated, in our Results Section 5 we refer to (i) “ideal cumulenic cases” with BLA =0, i.e., constant distance *d* between carbon atoms, and (ii) “ideal polyynic cases” with BLA ≠0, i.e., with alternating long $d_{\ell }$ and short $d_{s}$ distances, where all $d_{\ell }$ are constant and all $d_{s}$ are constant, but $d_{s}\neq d_{\ell }$. Specifically, we take d= 128.2 pm for the “ideal cumulenic molecules” and $d_{\ell }=$ 130.1 pm, $d_{s}=$ 126.5 pm for the “ideal polyynic molecules”, respectively, following Ref. [22].

## 3. Tight-Binding Wire Model Variants

Examples of atom sequences are shown in Figure 1. The carbon atom chain lies along the *z*-axis, which is collinear with $sp_{z}sp_{z}\sigma$ bonds (supposing sp hybridization between 2s and $2p_{z}$ carbon atomic orbitals). All $2p_{x}$ orbitals create a chain of ppπ interactions and, also, all $2p_{y}$ orbitals create a chain of ppπ interactions. Therefore, in a simplistic TB treatment, for either $2p_{x}$ orbitals or $2p_{y}$ orbitals, cumulenic carbynes can be regarded as a homogeneous chain with one electron per atom and one atom per unit cell, with a single on-site energy ϵ and a single hopping integral, *t*. Similarly, in a simplistic TB treatment, for either $2p_{x}$ orbitals or $2p_{y}$ orbitals, polyynic carbynes can be regarded as a chain with one electron per atom and two atoms per unit cell, with the same on-site energy ϵ and two hopping integrals, $t_{s}$ and $t_{\ell }$, for the short (*s*) and long (*l*) separations, respectively. Here we call this simplistic TB variant TBI. If there was no hybridization, if we would take into account all carbon valence orbitals (2s,$2p_{x}$,$2p_{y}$,$2p_{z}$), using the Slater–Koster [30] and the Harrison [31,32] expressions, a coarse estimate of the hopping integral between consecutive carbon atoms of separation *d* would be −0.36A, where $A=\frac{\hbar^{2}}{md^{2}}$. However, 2s and 2p orbitals are at different energies, hence, this view does not seem to hold. On the contrary, the carbyne chain is primarily formed by sp hybridizations, i.e, consecutive spspσ bonds, and secondarily allows for two ppπ sequences, one for $2p_{x}$ orbitals and one for $2p_{y}$ orbitals. If we think of a single ppπ chain, either for $2p_{x}$ orbitals or for $2p_{y}$ orbitals, then the hopping integral between consecutive carbon atoms of separation *d* would be $-0.63\frac{\hbar^{2}}{md^{2}}=-0.63A$, according to the Harrison expression [31,32]. This last choice makes TB approximately four times slower than RT-TDDFT in terms of frequency content of charge and dipole moment oscillations as well as in terms of coherent transfer rates [17]. In reality, the end sites are different than the middle sites because the carbon atoms there are connected with one or two or three hydrogen atoms, hence, their on-site energies as well as the hopping integrals between the first and the second site as well as between the penultimate and the ultimate site have to be modified. This modification, called TBImod, has been justified by qualitative arguments in Ref. [17]. A summary of TBImod parameters is given in Ref. [17]. Although TBImod predicts the same mean over time probabilities to find the hole at each site (site occupations) with RT-TDDFT, it is still four times slower in terms of frequency content of charge and dipole moment oscillations as well as in terms of coherent transfer rates [17].

To achieve, using a simple TB wire model, not only the same mean over time probabilities to find the hole at each site (site occupations) with RT-TDDFT, but also similarly fast frequency content of charge and dipole moment oscillations as well as coherent transfer rates, we have to multiply the TBImod transfer integrals by a factor of four [17]. We call this TB wire model TBImodt4times. We will try now to justify it qualitatively. The key is to realize that by forcing via CDFT (cf. Section 4) a hole to be created initially at the first site, we create this hole over all the first site orbitals and not at a specific orbital, e.g., the $2p_{x}$ or the $2p_{y}$ orbital. Hence, the propagation of the hole will proceed via all available channels and there are, approximately, three main channels. One is the spspσ channel, one is the ppπ ($2p_{x}$) channel and one is the ppπ ($2p_{y}$) channel. For the spspσ channel, occurring at an energy $\frac{E_{2s}C+E_{2p}C}{2}$, the estimation is the following. The left (L) and the right (R) sp orbitals can be written as

(1) $$|{sp}_{L}\rangle =\frac{1}{\sqrt{2}}(|s\rangle -|p\rangle ),\;|{sp}_{R}\rangle =\frac{1}{\sqrt{2}}(|s+p\rangle ).$$

Then, the transfer integral between two consecutive sites *i* and *j* would be

(2) $$\langle {sp}_{\mathrm{Ri}}\left|\hat{H}\right|{sp}_{\mathrm{Lj}}\rangle =\frac{1}{2}\langle s_{\mathrm{Ri}}\left|\hat{H}\right|s_{\mathrm{Lj}}\rangle -\frac{1}{2}\langle s_{\mathrm{Ri}}\left|\hat{H}\right|p_{\mathrm{Lj}}\rangle +\frac{1}{2}\langle p_{\mathrm{Ri}}\left|\hat{H}\right|s_{\mathrm{Lj}}\rangle -\frac{1}{2}\langle p_{\mathrm{Ri}}\left|\hat{H}\right|p_{\mathrm{Lj}}\rangle ,$$

which is equal to $\frac{A}{2}\left(-1.32-1.42-1.42-2.22\right)=\frac{A}{2}\left(-6.38\right)=-3.19A$. We have used the Harrison expressions [31,32] and taken into account the orientation of the constituent atomic orbitals. For each of the two ppπ channels made of $2p_{x}$ and $2p_{y}$ orbitals, respectively, the transfer integral between two consecutive sites *i* and *j* would be −0.63A. These channels occur at a higher energy compared to the previous channel, that is $E_{2p}C$, hence they will somehow have a higher weight relative to the spspσ channel, whereas, if we add up all these contributions we get −4.45A, which is very close to −4A, the necessary transfer integral between intermediate sites to bring the TB wire results very close to the RT-TDDFT results in terms of frequency content of charge and dipole moment oscillations as well as in terms of coherent transfer rates.

Although RT-TDDFT is a much more detailed method than TB (cf., Section 4), a comparison of the two in terms of computational cost is undoubtedly in favor of the latter. While a typical RT-TDDFT computation for systems of the size studied here needs several hours/days to be carried out in a computer cluster, the respective TB computation needs just some seconds to be carried out in a normal pc or laptop. As mentioned above, TBI and TBImod can reproduce some of our RT-TDDFT results, but cannot grasp the fast dynamics of charge transfer. On the other hand, TBImod4times passes that test as well, while still maintaining the advantage of computational efficiency over RT-TDDFT.

The Hamiltonian matrix in our TB wire model variants, for cumulenic molecules, has the form:

(3) $$H=[\begin{array}{cccccccc} \epsilon^{\prime } & t^{\prime } & & & & & & \\ t^{\prime } & \epsilon & t & & & & & \\ & t & \epsilon & t & & & & \\ & & t & \epsilon & t & & & \\ & & & \ddots & \ddots & \ddots & & \\ & & & & t & \epsilon & t & \\ & & & & & t & \epsilon & t^{\prime \prime }\\ & & & & & & t^{\prime \prime } & \epsilon^{\prime \prime } \end{array}]$$

In TBI, $\epsilon^{\prime }=\epsilon =\epsilon^{\prime \prime }$ and $t^{\prime }=t=t^{\prime \prime }$. Details and discussions of various aspects of the TBI wire model can be found elsewhere [5,33,34,35,36,37]. In TBImod, $\epsilon^{\prime }\neq \epsilon \neq \epsilon^{\prime \prime }$ and $t^{\prime }\neq t\neq t^{\prime \prime }$. For polyynic molecules, *t* become alternating $t_{s}$, $t_{l}$. The values of $\epsilon^{\prime },\epsilon ,\epsilon^{\prime \prime }$ and $t^{\prime },t,t^{\prime \prime }$ are given in Ref. [17]. In TBImodt4times, we multiply the hopping integrals of TBImod by 4.

## 4. Real-Time Time-Dependent Density Functional Theory

Density Functional Theory (DFT) [38,39] is an established method to obtain the ground state properties of molecules or solids. It has also been extended [40] to time-dependent systems (TDDFT). The Time-Dependent Kohn–Sham (TDKS) equations with an effective potential energy $\upsilon_{\mathrm{KS}}\left(r,t\right)$, uniquely described by a charge density ρ(r,t) are, in atomic units,

(4) $$\begin{array}{c} i\frac{\partial }{\partial t}\Psi_{j}\left(r,t\right)=[-\frac{1}{2}\nabla^{2}+\upsilon_{\mathrm{KS}}\left(r,t\right)]\Psi_{j}\left(r,t\right)=\\ {}[-\frac{1}{2}\nabla^{2}+\upsilon_{\mathrm{ext}}\left(r,t\right)+\upsilon_{H}\left(r,t\right)+\upsilon_{\mathrm{xc}}\left[\rho \right]\left(r,t\right)]\Psi_{j}\left(r,t\right). \end{array}$$

The charge density is the sum over all occupied orbitals $j=1,2,\cdots N_{occ}$, i.e.,

(5) $$\rho \left(r,t\right)=\sum_{j=1}^{N_{occ}}{\left|\Psi_{j}\left(r,t\right)\right|}^{2}.$$

The external fields and nuclear potentials are included in $\upsilon_{\mathrm{ext}}\left(r,t\right)$, $\upsilon_{H}\left(r,t\right)$ is the Hartree term, and $\upsilon_{\mathrm{xc}}\left[\rho \right]\left(r,t\right)$ is the exchange-correlation term.

Real-Time TDDFT (RT-TDDFT) [41] is based on direct numerical integration of Equation (4). The TDKS equations are solved at each time step, and the obtained electron density is subsequently used to calculate the Hamiltonian in the next step of the self-consistent cycle. For our DFT and RT-TDDFT calculations we used the NWChem open-source computational package [24], using typically a time step of 0.5 a.u..

The functional B3LYP [42,43,44,45] was used in all the results shown in this work. We have also tested CAM-B3LYP [46] without any major differences in the results. The calculations were performed using 3-21G [47] 6-31G* [48,49], cc-pVDZ [50], cc-pVTZ [50], and cc-pVQZ [50] (up to N=12) basis sets, for all systems. Increasing the size of the basis set, our results have clearly and nicely converged. At the beginning, the ground state of the neutral molecule was calculated by DFT, and the charge at each site was found. Then, the initial state was created with CDFT, putting everywhere the previously obtained charges, apart from the first site (CH or CH_2_ or CH_3_ group), where we increased the charge by +1, creating a hole). For example, for the cumulenic N=5 molecule, if we obtained at the beginning from DFT the charges +0.02,−0.01,−0.02,−0.01,+0.02, at CH_2_, C, C, C, CH_2_, respectively, then the CDFT constraints were +1.02,−0.01,−0.02,−0.01,+0.02, at CH_2_, C, C, C, CH_2_, respectively.

At the end of each time step, each fragment’s charge was calculated with an appropriate population analysis method, along with the dipole moment. We utilized Löwdin population analysis [51]. It was also used in the subsequent RT-TDDFT simulation. Löwdin population analysis was integrated by us into RT-TDDFT module of NWChem for the calculation of each fragment’s charge at each time step. It is much less basis-set dependent and does not suffer from ultra-fast oscillations that Mulliken analysis artificially introduces. (Mulliken analysis is the default scheme in NWChem’s RT-TDDFT). This way, we obtained a clearer picture of charge transfer. The main frequencies of charge and dipole moment oscillations are extracted via Fourier analysis.

## 5. Results

### 5.1. DFT Ground-State Energy

**Figure 6:**
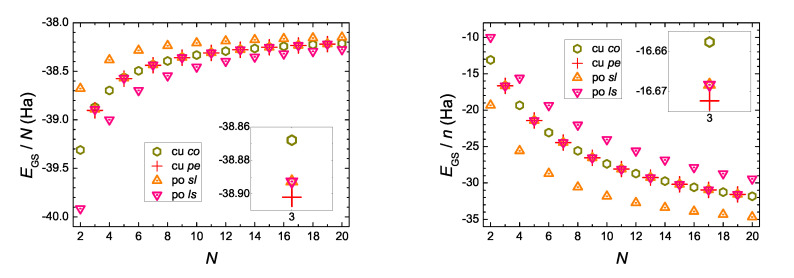
Ground state energy of neutral molecules, $E_{\mathrm{GS}}$, B3LYP/cc-pVTZ level of theory. *N* is the number of carbon atoms and *n* is the number of all atoms, cu *co* (cu *pe*) denotes cumulenic molecules with coplanar (perpendicular) methylene groups, po *sl* (po *ls*) denotes polyynic molecules starting with short (long) bonds. (**Left**) $E_{\mathrm{GS}}/N$ as a function of *N*. (**Right**) $E_{\mathrm{GS}}/n$ as a function of *N*. The insets emphasize that, for odd *N*, the cu *pe* (cu *co*) molecule has the lowest (highest) $E_{\mathrm{GS}}$.

In Figure 6 we present the ground-state energy, $E_{\mathrm{GS}}$, of neutral molecules at the B3LYP/cc-pVTZ level of theory. We observe that $\frac{E_{\mathrm{GS}}}{N}\left(N\right)$ are increasing functions of *N*, while $\frac{E_{\mathrm{GS}}}{n}\left(N\right)$ are decreasing functions of *N*. For even *N*, there are big differences between polyynic *sl*, cumulenic *co* and polyynic *ls* molecules, the cumulenic *co* is always in the middle, while, the order of polyynic molecules changes if we consider $E_{\mathrm{GS}}/n$ instead of $E_{\mathrm{GS}}/N$. For odd *N*, the $E_{\mathrm{GS}}$ of all molecules are much closer than for even *N*. For odd *N*, the cumulenic *pe* molecule, which exists only for odd *N*, is, in fact, the molecule with the lowest $E_{\mathrm{GS}}$, while the cumulenic *co* version is the molecule with the highest $E_{\mathrm{GS}}$ (cf. Figure 6 insets).

### 5.2. CDFT “Ground-State” Energy with a Hole at the First Site

**Figure 7:**
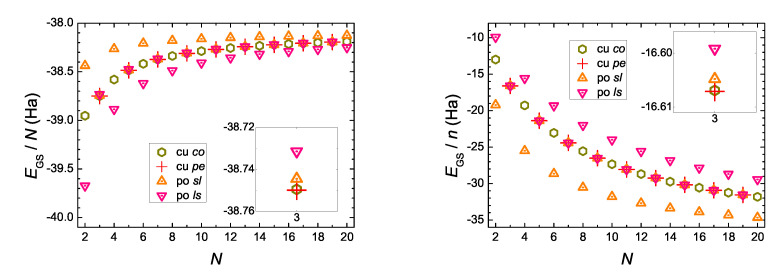
“Ground state” energy, $E_{\mathrm{GS}}$, of molecules with a hole created at its first site, B3LYP/cc-pVTZ level of theory. *N* is the number of carbon atoms, *n* is the number of all atoms, cu *co* and cu *pe* denote cumulenic molecules with coplanar and perpendicular methylene groups, respectively; po *sl* and po *ls* denote polyynic molecules starting with short and long bonds, respectively. The insets emphasize that, for odd *N*, the cu *pe* and cu *co* molecules come closer in energy (it seems that for N>3 cu *co* has slightly lower energy), po *sl* has higher energy and po *ls* still higher.

The creation of a hole at the first site (CH or CH_2_ or CH_3_) by CDFT affects the “ground-state” energy, depicted in Figure 7 in a similar way as the ground state energy of the neutral molecules is depicted in Figure 6. A molecule with a hole has larger energy than the respective neutral molecule, as expected. This is why neutral molecules exist. CDFT evaluates the excited state energy in accord with its constraint. Therefore, the term “ground-state” is excessive here. The insets illustrate that, for odd *N*, the creation of a hole brings the two cumulenic molecules with coplanar or perpendicular methylene groups much closer in energy (actually, it seems that for N>3 cumulenic *co* has slightly lower energy), the polyynic *sl* molecule (creation of a hole at CH) has slightly higher energy, and the creation of a hole at a polyynic *ls* molecule (creation of a hole at CH_3_) has even higher energy. These differences diminish increasing *N*, as expected.

### 5.3. Eigenenergies, Density of States, and Energy Gap

**Figure 8:**
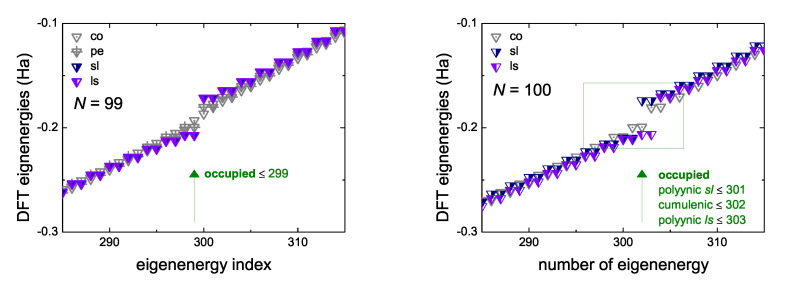
An example, for N=99 and N=100, of the formation of energy gap between occupied and empty eigenstates of neutral molecules, as obtained by our DFT simulations, at the B3LYP/cc-pVTZ level of theory.

**Figure 9:**
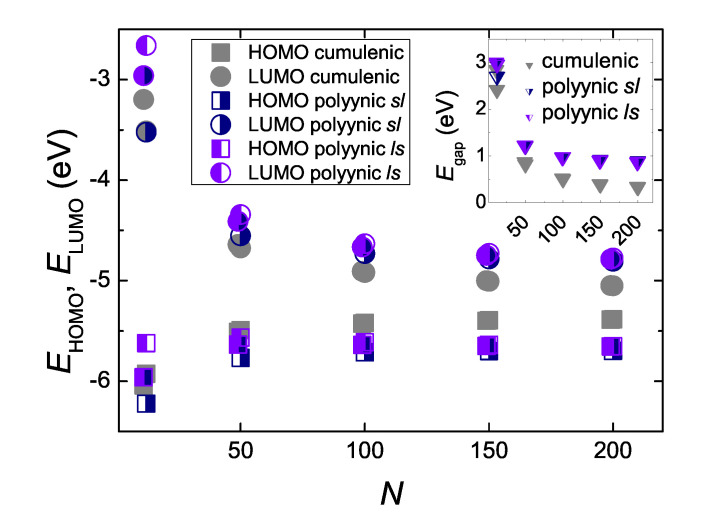
The eigenenergies of the Highest Occupied Molecular Orbital (HOMO), the Lowest Unoccupied Molecular Orbital (LUMO) and the energy gap (inset) between them, as functions of the number of carbon atoms in the chain, *N*, as obtained by our DFT simulations, at the B3LYP/cc-pVTZ level of theory. The points shown correspond to *N* = 11, 12, 49, 50, 99, 100, 149, 150, 199, 200.

In Figure 8 we give an example, for N=99 and N=100, where we observe the formation of an energy gap between occupied and empty eigenstates of neutral molecules, at the B3LYP/cc-pVTZ level of theory. For N=99, the cumulenic *co* molecule does not show an energy gap, but the cumulenic molecule with the lowest ground state energy, i.e., the cumulenic *pe* molecule, does. The conclusion is that cumulenes also have an energy gap, albeit smaller than polyynes. The evolution of the eigenenergies of the Highest Occupied Molecular Orbital (HOMO), $E_{\mathrm{HOMO}}$, the Lowest Unoccupied Molecular Orbital (LUMO), $E_{\mathrm{LUMO}}$, and of the energy gap between them, $E_{\mathrm{gap}}$, for cumulenes and polyynes, increasing *N*, are illustrated in Figure 9. Increasing *N*, the energy gap approaches ≈ 0.3 eV for cumulenes and ≈ 0.9 eV for polyynes.

**Figure 10:**
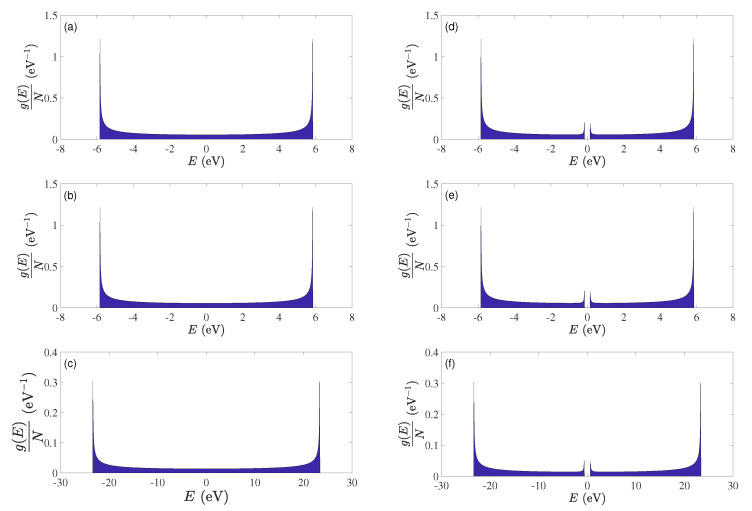
Density of states, g(E), per number of carbon atoms, *N*, for the three Tight-Binding (TB) variants used in this work. (**a**) cumulenic TBI, (**b**) cumulenic TBImod, (**c**) cumulenic TBImodt4times, (**d**) polyynic TBI, (**e**) polyynic TBImod, (**f**) polyynic TBImodt4times.

Within the simplistic TBI approach, cumulenic and polyynic carbynes are mathematically equivalent to type $\alpha^{\prime }$ and type $\beta^{\prime }$ polymers, respectively. Details of these can be found in Ref. [34]. It can be proved analytically that cumulenes do not have an energy gap, while, the energy gap in the polyynic case is equal to $2|t_{s}-t_{l}|$. Since $t_{l}=-2.84$ eV and $t_{s}=-3.00$ eV, $E_{\mathrm{gap}}=0.32$ eV. The TB eigenspectra, density of states, and energy gap for TBI, TBImod and TBImodt4times have been also commented in Ref. [17]. Here we present in Figure 10 the density of states for the three TB variants used in this work, for cumulenic and polyynic carbynes. In contrast to DFT, in TB cumulenes do not show an energy gap, while polyynes show an energy gap of the order ≈ 0.3 eV (TBI, TBImod) or ≈1.2 eV (TBImodt4times).

### 5.4. Charge Oscillations

**Figure 11:**
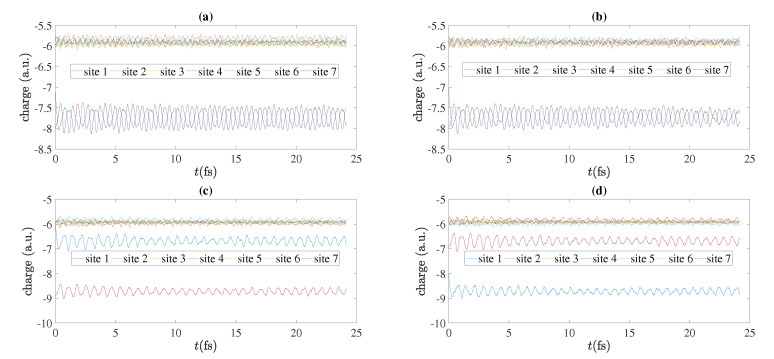
Charge oscillations obtained by RT-TDDFT, at the B3LYP/cc-pVTZ level of theory, N=7, for cumulenic (*cu*) molecules with coplanar (*co*) and perpendicular (*pe*) methylene groups as well as polyynic (*po*) molecules starting with short (*sl*) or long (*ls*) bonds. (**a**) *cu co*, (**b**) *cu pe*, (**c**) *po sl*, (**d**) *po ls*.

**Figure 12:**
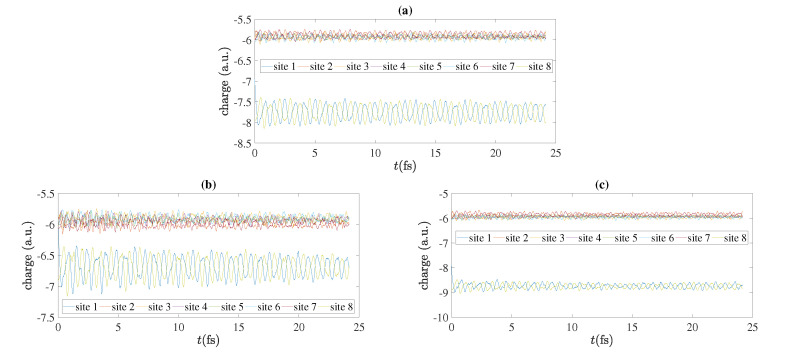
Charge oscillations obtained by RT-TDDFT, at the B3LYP/cc-pVTZ level of theory, N=8, for cumulenic (*cu*) molecules with coplanar (*co*) methylene groups as well as polyynic (*po*) molecules starting with short (*sl*) or long (*ls*) bonds. (**a**) *cu co*, (**b**) *po sl*, (**c**) *po ls*.

As an example, we present in Figure 11 charge oscillations obtained by RT-TDDFT, for N=7, for cumulenic molecules with coplanar or perpendicular methylene groups and for polyynic molecules starting with short or long bonds, at the B3LYP/cc-pVTZ level of theory. We show the total electronic charge at each site as a function of time. Without placing a hole, a C site contains six electrons, a CH site contains seven electrons, a CH_2_ site contains eight electrons and a CH_3_ site contains nine electrons. We place the hole initially (time zero) at the first site, as always in this article. In Figure 12 we show charge oscillations obtained by RT-TDDFT, for N=8, for cumulenic molecules with coplanar methylene groups and for polyynic molecules starting with short or long bonds, at the B3LYP/cc-pVTZ level of theory.

**Figure 13:**
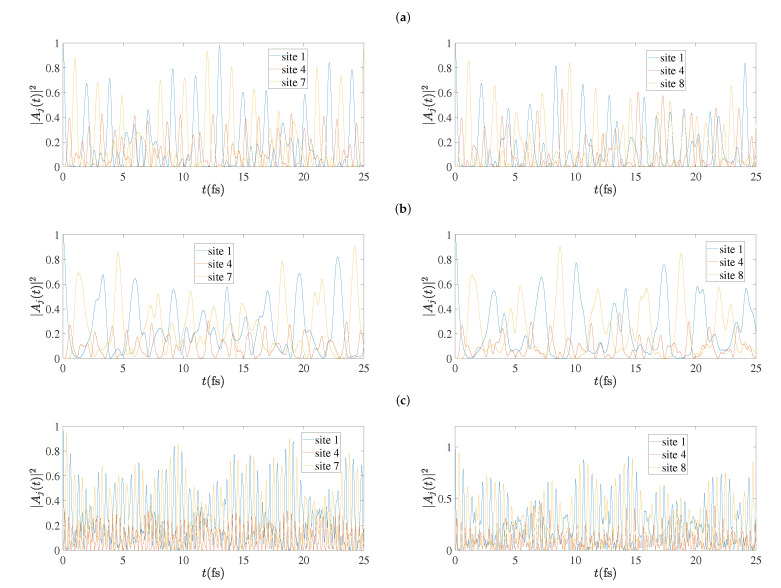
Charge oscillations obtained by the TB variants (**a**) TBI, (**b**) TBImod, (**c**) TBImodt4times, for cumulenic molecules with N=7 (left column) and N=8 (right column).

In Figure 13 we present the time-dependent probabilities to find the hole at each site, $|A_{j}{\left(t\right)|}^{2}$, as obtained by the TB variants for cumulenic molecules with N=7 (left column) and N=8 (right column). The RT-TDDFT dynamics is faster than TBI and TBImod dynamics, although the mean over-time probabilities at each site (cf. Section 5.5) are very close for TBImod and RT-TDDFT. In other words, TBI and TBImod cannot follow the fast dynamics of RT-TDDFT. This is also evident in Section 5.6, Section 5.7 and Section 5.8. On the contrary, the TBImodt4times dynamics is similarly fast with the RT-TDDFT dynamics.

### 5.5. Mean over Time Probabilities

**Figure 14:**
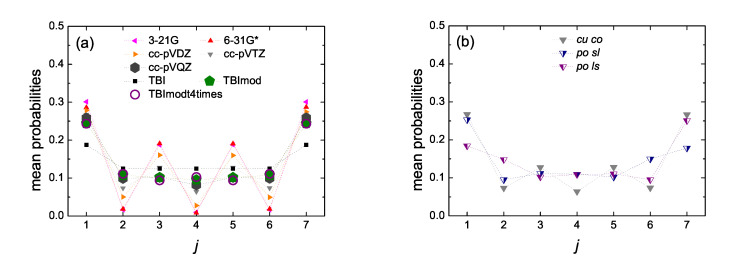

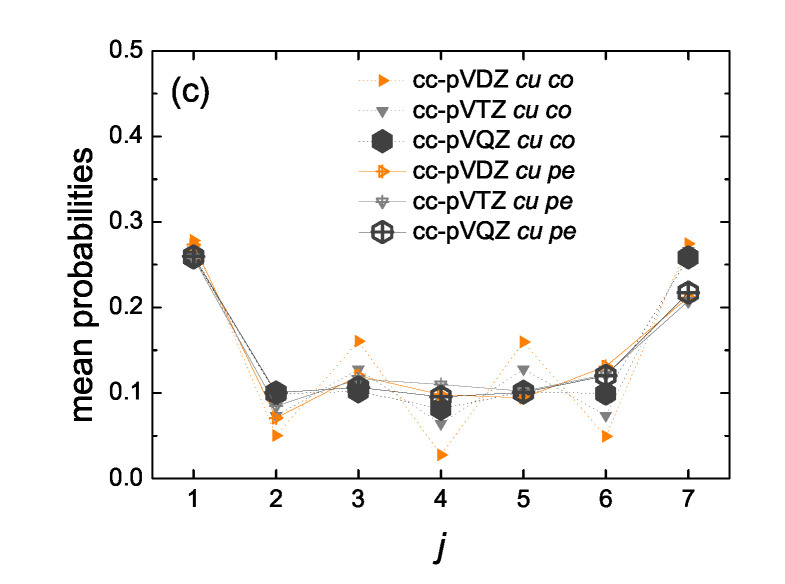
Site occupations, i.e., mean over time probabilities to find the hole at site *j*, for initial placement of the hole at the first site, for N= 7, obtained by Real-Time Time-Dependent Density Functional Theory (RT-TDDFT) [3-21G (pink left triangles), 6-31G* (red up triangles), cc-pVDZ (orange right triangles), cc-pVTZ (gray down triangles), cc-pVQZ (dark gray hexagons)] and the functional B3LYP, as well as by TB wire model variants [TBI (black squares), TBImod (green pentagons), TBImodt4times (purple circles)]. (**a**) cumulenic molecules with coplanar methylene groups (*cu co*), (**b**) cc-pVTZ/B3LYP for *cu co* molecules versus polyynic molecules starting with short or long bonds (*po sl* or *po ls*). Half-filled down triangles for *po sl* (blue filled right) and *po ls* (purple filled left). (**c**) *cu co* versus cumulenic molecules with perpendicular methylene groups (*cu pe*) for the 3 larger basis sets. Dotted lines are guides to the eyes.

**Figure 15:**
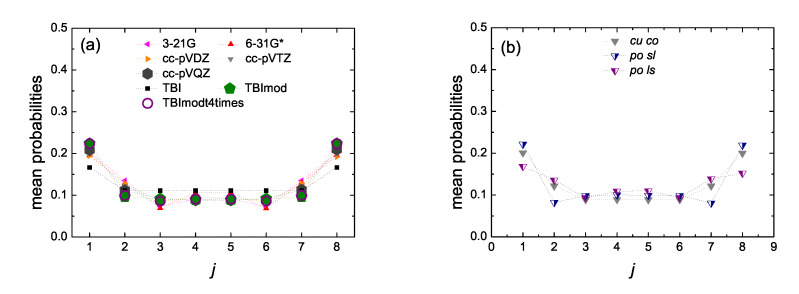
Site occupations, i.e., mean over time probabilities to find the hole at site *j*, for initial placement of the hole at the first site, for N= 8, obtained by RT-TDDFT [3-21G (pink left triangles), 6-31G* (red up triangles), cc-pVDZ (orange right triangles), cc-pVTZ (gray down triangles), cc-pVQZ (dark gray hexagons)] and functional B3LYP as well as by TB wire model variants [TBI (black squares), TBImod (green pentagons), TBImodt4times (purple circles)]. (**a**) cumulenic molecules with coplanar methylene groups (*cu co*), (**b**) cc-pVTZ/B3LYP for *cu co* molecules versus polyynic molecules starting with short or long bonds (*po sl* or *po ls*). Half-filled down triangles correspond to *po sl* (blue filled right) and *po ls* (purple filled left). Dotted lines are guides to the eyes.

In Figure 14 and Figure 15 we present the mean over time probabilities to find the hole at each site *j*, having placed it initially at the first site, for open cumulenic and polyynic carbynes, for N= 7 and 8, respectively. The results shown were calculated by RT-TDDFT, using the basis sets 3–21 G, 6–31 G*, cc-pVDZ, cc-pVTZ, cc-pVQZ and the functional B3LYP. We also show the TBI, TBImod, TBImodt4times results, for comparison. We compare cumulenic (*cu*) molecules with coplanar (*co*) or perpendicular (*pe*) methylene groups as well as polyynic (*po*) molecules starting with short (*sl*) or long (*ls*) bonds. Increasing the size of the basis set, we observe the clear convergence of the RT-TDDFT results as well as the convergence of the RT-TDDFT results with the TB variants, increasing the level of TB elaboration. Finally, we observe the little differences in site occupations between *cu co*, *cu pe*, *po sl*, *po ls* molecules. A short discussion about these differences can be found in Ref. [17].

### 5.6. Coherent Transfer Rates

Coherent transfer rates can be described by the *pure* mean transfer rate [33], defined by

(6) $$k_{\lambda \mu }=\frac{\langle |C_{\mu }\left(t\right){|}^{2}\rangle }{t_{\lambda \mu }}.$$

**Figure 16:**
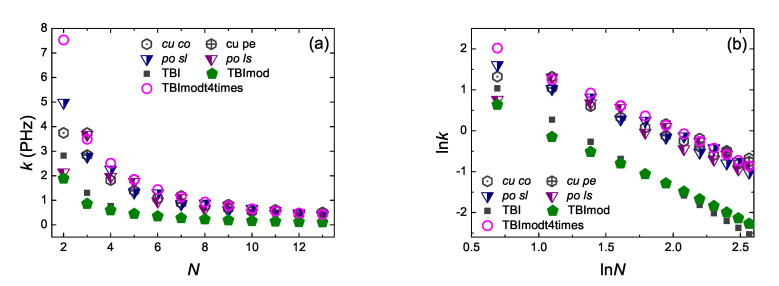
Transfer rates along carbyne wires as obtained by RT-TDDFT at the B3LYP/cc-pVTZ level of theory as well as by TBI, TBImod and TBImodt4times: *cu co* (dark gray hexagons with dot), *cu pe* (dark gray hexagons with cross), *po sl* (blue right half-filled down triangles), *po ls* (purple left half-filled down triangles), TBI (black squares), TBImod (olive pentagons), TBImodt4times (magenta circles). (**a**) k(N), (**b**) lnk(lnN).

Here $t_{\lambda \mu }$ is the mean transfer time, i.e., the necessary time for the probability to find the extra carrier at site μ, $|C_{\mu }\left(t\right){|}^{2}$, to become equal to its mean value, $\langle |C_{\mu }\left(t\right){|}^{2}\rangle$, for the first time, having placed at time zero the carrier at site λ. Defined this way, *k* evaluates the magnitude of charge transfer and the time scale of the phenomenon. A comparison of k(N) and lnk(lnN) for initial hole placement at the first site and up to the last site ($k_{1N}:=k$), as obtained by RT-TDDFT at the B3LYP/cc-pVTZ level of theory as well as by the TB variants TBI, TBImod, TBImodt4times, is shown in Figure 16. Although RT-TDDFT is followed by TBI and –slightly better– TBImod in a parallel natural logarithmic manner, the two latter cannot reproduce quantitatively the fast dynamics of RT-TDDFT. However, the transfer rates obtained by TBImodt4times follow closely the transfer rates obtained by RT-TDDFT. A discussion and comparison of the transfer rates of cumulenic vs. polyynic carbynes, obtained by RT-TDDFT, can be found in Ref. [17].

### 5.7. Electric Dipole Moment

**Figure 17:**
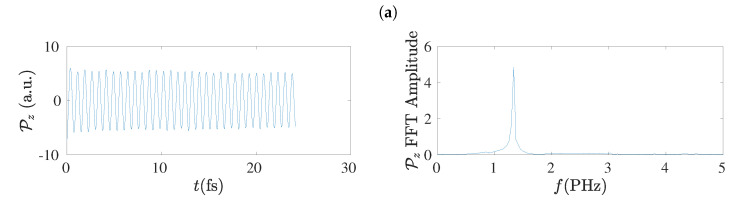

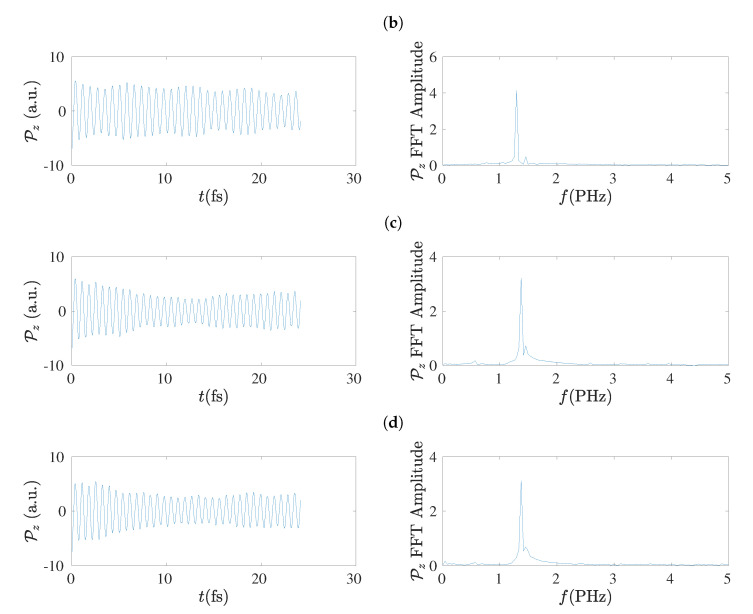
Left column: Dipole moment oscillations along the *z*-axis, $P_{z}$, obtained by RT-TDDFT at the B3LYP/cc-pVTZ level of theory, for N=7, for cumulenic (*cu*) molecules with coplanar (*co*) and perpendicular (*pe*) methylene groups as well as for polyynic (*po*) molecules starting with short (*sl*) or long (*ls*) bonds. Right column: The corresponding Fast Fourier Transform (FFT) amplitudes obtained by MATLAB. (**a**) *cu co*, (**b**) *cu pe*, (**c**) *po sl*, (**d**) *po ls*.

**Figure 18:**
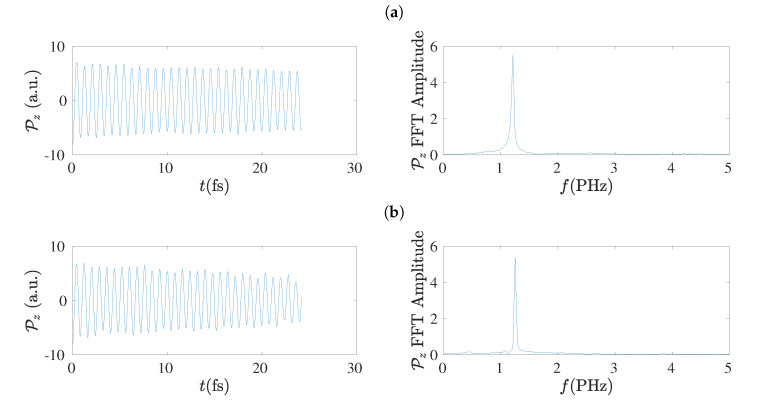

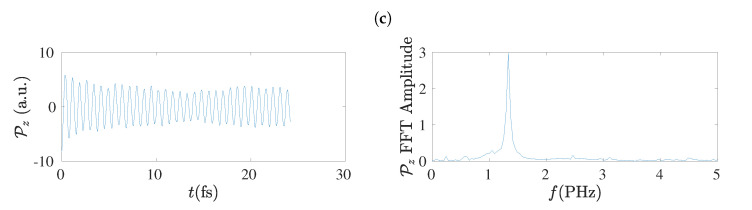
Left column: Dipole moment oscillations along the *z*-axis, $P_{z}$, obtained by RT-TDDFT at the B3LYP/cc-pVTZ level of theory, for N=8, for cumulenic (*cu*) molecules with coplanar (*co*) methylene groups as well as for polyynic (*po*) molecules starting with short (*sl*) or long (*ls*) bonds. Right column: The corresponding FFT amplitudes obtained by MATLAB without any further elaboration. (**a**) *cu co*, (**b**) *po sl*, (**c**) *po ls*.

The electric dipole moment, P, has the advantage of being independent on the population (charge) analysis. Therefore, it can be used to extract the frequency content of hole oscillations [17] without concern that charge analysis might have some influence. In the left columns of Figure 17 and Figure 18 we give examples of the electric dipole moment oscillations along the *z*-axis, for molecules with N=7 and N=8, respectively, obtained by RT-TDDFT at the B3LYP/cc-pVTZ level of theory. In these figures we omit dipole moment oscillations along the *x*-axis and the *y*-axis, because their maximum values are of the order of $10^{-6}$ to $10^{-7}$ a.u. for cumulenes (both end groups CH_2_–) and even *N* *sl* polyynes (both end groups CH–), and of the order of 0.1 a.u. for other polyynes. In the right columns of Figure 17 and Figure 18 we present the corresponding FFT of each $P_{z}$, as obtained simply by MATLAB, without any further elaboration. The time step in RT-TDDFT was 0.5 a.u.; we covered 1000 a.u. ≈ 25 fs with ≈ 2000 points.

**Figure 19:**
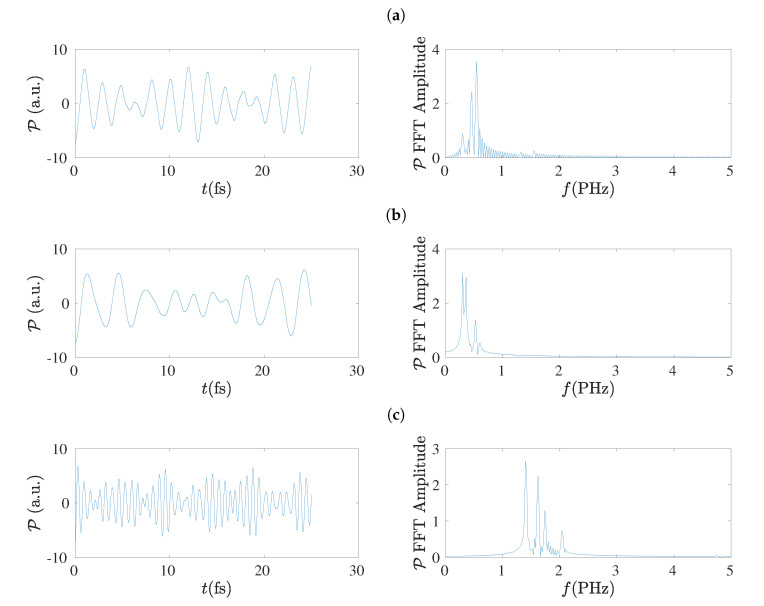
Left column: Dipole moment (P) oscillations obtained by the TB variants (**a**) TBI, (**b**) TBImod, (**c**) TBImodt4times, for N=7. Right column: The corresponding FFT amplitudes obtained by MATLAB without any further elaboration.

**Figure 20:**
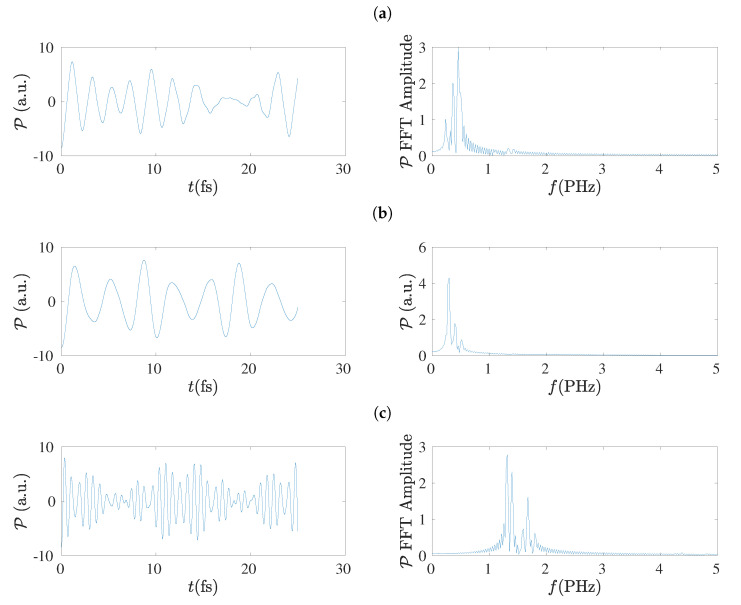
Left column: Dipole moment (P) oscillations obtained by the TB variants (**a**) TBI, (**b**) TBImod, (**c**) TBImodt4times, for N=8. Right column: The corresponding FFT amplitudes obtained by MATLAB without any further elaboration.

In the left columns of Figure 19 and Figure 20 we present the dipole moment (P) oscillations as obtained by the TB variants TBI, TBImod and TBImodt4times, for molecules with N=7 and N=8, respectively, and in their right columns the corresponding FFT of each P, as obtained simply by MATLAB, again without any further elaboration. The number of points in the TB simulations for the duration of 25 fs is 128 × 16,385. The main conclusion from these figures is that TBI and TBImod produce dipole moment oscillations not fast enough to compete with RT-TDDFT. However, TBImodt4times dipole moment oscillations have similar frequency content with RT-TDDFT.

### 5.8. Frequency Content

Using the dipole moment oscillations, simply by FFT, the frequency content of the oscillations can be obtained [17], as shown also in Section 5.7. The summary is that TBI and TBImod can not follow the fast dynamics of RT-TDDFT and in order to obtain by TB similar frequency content with that of RT-TDDFT, we have to employ TBImodt4times. The FFT spectrum is slightly influenced by varying the time of simulation, but the picture does not change qualitatively. The main frequency is in the PHz range and it falls with increasing *N*, as expected.

## 6. Conclusions

We have studied open cumulenes and polyynes using DFT, CDFT, RT-TDDFT and TB variants.

We have clearly obtained converging results using the functional B3LYP and the basis sets 3-21G, 6-31G*, cc-pVDZ, cc-pVTZ, cc-pVQZ in terms of all the studied physical properties, including ground-state energy, energy gap between occupied and empty eigenstates, site occupations, coherent transfer rates, charge and dipole moment oscillations and frequency content in general.

We have also utilized three TB wire models: a simplistic model where all sites are equivalent and the transfer integrals are given by the Harrison ppπ expression (TBI) as well as a model with same transfer integrals but with modified initial and final sites (TBImod) to allow for the existence of one or two or three hydrogen atoms at the edge sites. To achieve, in cumulenes, similar site occupations with the converged RT-TDDFT ones, TBImod is sufficient. However, to achieve similar frequency content of charge and dipole moment oscillations and similar coherent transfer rates, the TBI, TBImod transfer integrals have to be multiplied by a factor of four (TBImodt4times). We gave an explanation for this fact. Briefly, the reason is that in CDFT the hole is created at the first site and not at a specific orbital of the first site, hence, there are approximately three channels for charge transfer: one spspσ channel (sp hybridized 2s and $2p_{z}$ orbitals), one ppπ ($2p_{x}$) channel and one ppπ ($2p_{y}$) channel. Evaluating the different coupling strengths of these channels, we estimated a factor of approximately four relative to the Harrison ppπ expression.

Full geometry optimization at the B3LYP/6-31G* level of theory has showed that in cumulenes BLA is not strictly zero and is not constant, although it is symmetrical relative to the molecule center. BLA in cumulenic cases is much smaller than in polyynic cases, so, although not strictly, the separation to cumulenes and polyynes, approximately, holds. Cumulenes have longer C–C bonds at the ends (≈132 pm) and C–C bond lengths ≈127 to 129 pm at the middle with alternating longer and shorter bonds. In this sense, the resulting geometry is not strictly cumulenic, in the sense that BLA ≠0 and is not constant, but it is symmetrical relative to the molecule center. In cumulenes with *N* odd, *pe* molecules have shorter bond lengths than *co* molecules, which is another manifestation that their ground state energy is lower. Polyynes with *N* odd have one possible geometry, *sl*≡ *ls*, but polyynes with *N* even have three possible geometries, *sl*, *lss* and *lse*; *lss* and *lse* denote staggered and eclipsed methyl end groups with negligible difference in their ground state energies. Polyynes have long bonds at the ends (≈145 pm) which are longer than the long bonds in the middle (≈135 pm), and short bonds ≈122 pm.

Vibrational analysis has confirmed that for *N* even all cumulenes with coplanar methylene end groups are stable, for *N* odd all cumulenes with perpendicular methylene end groups are stable, and the number of hydrogen atoms at the end groups is clearly seen in all cumulenic and polyynic cases as higher frequencies.

We have calculated and discussed the DFT ground state energy of neutral molecules, the CDFT “ground state energy” of molecules with a hole at one end group, energy spectra, density of states, energy gap, charge and dipole moment oscillations, site occupations, coherent transfer rates, and the frequency content, in general. We have also compared RT-TDDFT with TB results.

Concerning the ground-state energy: For even *N*, $E_{\mathrm{GS}}$ of *po sl*, *cu co* and *po ls* molecules is clearly different; $E_{\mathrm{GS}}$ of *cu co* is always in the middle; the order of *po sl* and *po ls* changes if we consider $E_{\mathrm{GS}}/n$ instead of $E_{\mathrm{GS}}/N$. For odd *N*, $E_{\mathrm{GS}}$ of all molecules is much closer; the *cu pe* molecule is the one with the lowest $E_{\mathrm{GS}}$, while the *cu co* is the molecule with the highest $E_{\mathrm{GS}}$.

The creation of a hole by CDFT at the initial site leads to larger energy than the respective neutral molecule, as expected. For odd *N*, the creation of a hole brings *cu co* and *cu pe* molecules much closer in energy; the *po sl* molecule has slightly higher energy, and the *po ls* molecule has even higher energy. These differences diminish increasing *N*, as expected.

DFT shows that cumulenes also have an energy gap between occupied and empty states, smaller than polyynes. At the limit of large *N*, the energy gap approaches ≈0.3 eV for cumulenes and ≈0.9 eV for polyynes. In TB cumulenes do not show an energy gap, while polyynes show an energy gap of the order ≈ 0.3 eV (TBI, TBImod) or ≈ 1.2 eV (TBImodt4times).

Concerning charge oscillations, dipole moment oscillations and the frequency content in general as well as concerning coherent transfer rates, RT-TDDFT dynamics is faster than TBI and TBImod dynamics, although the mean over-time probabilities at each site are very close for TBImod and RT-TDDFT. TBImodt4times which uses the same on-site energies with TBImod but transfer integrals four times larger, can follow the fast dynamics of RT-TDDFT in terms of all the above mentioned quantities, keeping at the same time mean over-time probabilities at each site very close to the converged RT-TDDFT ones, and having a significantly less computational cost than RT-TDDFT. 

## Figures and Tables

**Table 1:** Carbon–carbon bond lengths *d* (pm) in organic compounds.

	*d*		*d*		*d*		*d*		*d*
$sp^{3}$-$sp^{3}$	154 [19]	$sp^{3}$-sp	146 [19]	C−C	154 [20,21]	benzene	140 [19]	polyynic long	130.1 [22]
$sp^{3}$-$sp^{2}$	150 [19]	$sp^{2}$-sp	143 [19]	C=C	134 [20,21]	alkene	134 [19]	cumulenic	128.2 [22]
$sp^{2}$-$sp^{2}$	147 [19]	sp-sp	137 [19]	C≡C	120 [20,21]	alkyne	120 [19]	polyynic short	126.5 [22]

**Table 2:** *n* is the number of atoms, *N* is the number of carbon atoms. The number of modes, m=3n, from which three are translational modes (TM). Linear and nonlinear molecules have two and three rotational modes (RM), respectively; therefore, the number of vibrational modes (VM) is 3n−5 and 3n−6, respectively.

Type	*N*	*n*	TM	RM	VM
cumulenes	even, odd	N+4	3	3	3n−6
polyynes *sl*	even	N+2	3	2	3n−5
polyynes *sl*	odd	N+4	3	3	3n−6
polyynes *ls*	even	N+6	3	3	3n−6
polyynes *ls*	odd	N+4	3	3	3n−6

## Abbreviations

The following abbreviations are used in this manuscript:

BLA	bond length alternation
CDFT	Constrained Density Functional Theory
DFT	Density Functional Theory
DOS	density of states
FFT	Fast Fourier Transform
RT-TDDFT	Real-Time Time-Dependent Density Functional Theory
TB	Tight Binding
TBI	TB wire model
TBImod	a crude modification of TB wire model
TBImodt4times	TBImod with four times greater transfer parameters
TDDFT	time-dependent DFT
TDKS	time-dependent Kohn-Sham
